# The association between FTO rs9939609 polymorphism and serum lipid profile in adult women

**DOI:** 10.1186/s13098-021-00754-0

**Published:** 2021-11-20

**Authors:** Vahideh Jalili, Zohreh Mokhtari, Samira Rastgoo, Azadeh Hajipour, Fatemeh Bourbour, Maryam Gholamalizadeh, Alireza Mosavi Jarrahi, Sepehr JavadiKooshesh, Alireza Moslem, Morteza Abdollahi, Saeid Doaei

**Affiliations:** 1 Faculty of Medicine, Urmia University of Medical Sciences, Urmia, Iran; 2 Department of Clinical Biochemistry, Faculty of Medicine, Shahid Beheshti University of Medical Sciences, Tehran, Iran; 3 National Nutrition and Food Technology Research Institute, Faculty of Nutrition Sciences and Food Technology, Shahid Beheshti University of Medical Sciences, Tehran, Iran; 4 School of Health, Qazvin University of Medical Sciences, Qazvin, Iran; 5 Student Research Committee, Cancer Research Center, Shahid Beheshti University of Medical Sciences, Tehran, Iran; 6 School of Medicine, Shahid Beheshti University of Medical Sciences, Tehran, Iran; 7 Department of Medical Genetics, School of Medicine, Shahid Beheshti University of Medical Sciences, Tehran, Iran; 8 Department of Anesthesiology, Sabzevar University of Medical Sciences, Sabzevar, Iran; 9 Department of Nutrition Research, National Nutrition and Food Technology Research Institute, School of Nutrition Sciences and Food Technology, Shahid Beheshti University of Medical Sciences, Tehran, Iran; 10 Reproductive Health Research Center, Department of Obstetrics and Gynecology, Al-zahra Hospital, School of Medicine, Guilan University of Medical Sciences, Rasht, Iran

**Keywords:** FTO gene, Serum lipids, Polymorphism

## Abstract

**Background:**

FTO gene is considered to play an important role in many metabolic diseases. Evidence from studies indicated the possible association between the FTO rs9939609 polymorphisms with serum lipid profile. Therefore, this study aimed to investigate the association of FTO rs9939609 polymorphism with lipid profile in Iranian women.

**Methods:**

This cross-sectional study was carried out on 380 adult women. Information about age, height, weight, BMI, physical activity, and dietary intake were collected. The serum levels of Low Density Lipoprotein (LDL), High Density Lipoprotein (HDL), Triglyceride (TG), and total cholesterol were measured. The FTO gene was genotyped for rs9939609 polymorphism. The participants were divided into two groups of TT and AT/AA considering dominant model of FTO rs9939609 polymorphism.

**Results:**

General characteristics of the participants with different FTO genotypes were not significantly different. The lower levels of HDL were observed in AT/AA genotypes compared to the TT wild type genotype of FTO rs9939609 polymorphism (P = 0.004). Adjustments of age, BMI, and physical activity did not change the results.

**Conclusions:**

However, the significant association between FTO genotype and the HDL level was disappeared after further adjustments for dietary intake. Further studies are warranted to identify the underlying mechanisms of the possible association between FTO gene and serum lipid profile.

## Introduction

The recent epidemics of chronic diseases have largely been attributed to the genetic background and changes in lifestyle [1]. The prevalence of metabolic syndrome (MS) increasing around the world [2]. Women in the Middle East (25%) and north Africa have the highest risk of metabolic diseases of all women globaly [3, 4]. In East Asian countries, the prevalence of MS was ranged from 2 to 18% in women and from 8 to 13% in men [5] According to statistics in Iran, the prevalence of MS among adolescents is more than 30% [6]. MS and other cardio metabolic disorders including obesity, type 2 diabetes (T2D), and cardiovascular disease (CVD) are associated with dysregulation of lipid metabolism [7, 8]. Elevated levels of total cholesterol (TC), triglycerides (TG), and low-density lipoprotein cholesterol (LDL-C), and decreased level of high-density lipoprotein cholesterol (HDL-C) are used as biomarkers of the chronic diseases [8, 9]. MS is reported to be more frequent in women (25.3%) than in men (23.2%) and the most frequent component of MS was low HDL cholesterol, which was reported in 62.9% of cases in 2009 [10] and is mainly caused by genetics and lifestyle factors [11].

Defining the possible association between lipid profile and gene polymorphisms helps to identify potential biomarkers associated with the risk of MS and to understand the genetic basis of cardio metabolic diseases [12, 13]. FTO protein is widely expressed in the hypothalamus, which is critical for control of energy homeostasis and eating behavior [14, 15] Different studies indicated that the regulation of FTO mRNA expression is related to food intake [14, 16, 17], blood glucose level [18], body weight [19], and energy consumption [20, 21]. The FTO gene is reported to have key roles in the regulation of energy balance and lipid metabolic process pathways [22].

Although, different studies on various populations were performed on the relation of the FTO variations with lipid profile, there is still no agreement in this regard. The prevalence of abnormal lipid profile in Iran is increasing in both genders, with women having twofold higher prevalence of high total cholesterol than men aged ≥ 45 years [23] and this gender-dependent difference can be associated with FTO genotype [24]. So, this study aimed to investigate the association of FTO rs9939609 polymorphism with lipid profile only in Iranian women.

## Methods

### Study population and data collecting

This cross-sectional study was carried out from September 2018 to February 2019 on 380 adult Iranian women in Tehran, Iran. The sample size was calculated using odds ratio of a similar study [25]. The participations were selected from the Sabzevar study of Persian cohort using the randomized selection method. The subjects were included if they were between 35 to 75 years old, had no history of MS, and not using of cholesterol and lipid lowering drugs. The aims of the study were explained for the participants and the written consent forms were collected. The participants were excluded if their blood samples and/or the required data were not available (n = 5). Finally, the analysis was performed on 375 people. Information about age, height, weight, and BMI were collected through face to face interview. Physical activity was estimated using a validated International Physical Activity Questionnaires (IPAQ) [26]. The amount of dietary calorie and macro-nutrients intake were assessed by a validated food frequency questionnaire (FFQ) [27].

### Lipid profile measurement

5 ml of venous blood samples of participants were collected after 12 h of fasting to check lipid Prole including Low Density Lipoprotein (LDL), High Density Lipoprotein (HDL), Triglyceride (TG), and total cholesterol using photometric method and quantitative diagnostic kit (Parsazmoon Co., Tehran, Iran). Abnormal levels of TG, total cholesterol, HDL, and LDL were considered as 150 mg/dl, 200 mg/dl, 50 mg/dl, and 100, respectively (https://labtestsonline.org/test/lipid-panel).

### Genotyping

DNA was extracted from whole blood samples using the DNA extraction kit (gene all Co., South Korea). DNA samples were amplified using polymerase chain reaction (PCR) and master mix polymerase (Cat. No A180301; Ampliqon Denmark), and then the tetra-primer amplification refractory mutation system-PCR (TETRA ARMS-PCR) method was used for determining FTO rs9939609 polymorphism genotype.

### Statistical analysis

The participants were divided into two groups of TT and AT/AA considering dominant model of FTO rs9939609 polymorphism. Two groups were compared in terms of demographic and pathological factors using independent T- test and Qi-square methods. Logistic regression was then used to investigate the association of serum lipid profile with the risk allele of FTO rs9939609 polymorphism as crude (model 1), after adjustment of confounding variables including age and BMI (model 2), further adjustments for physical activity (model 3), and additional adjustments dietary intake (model 4). All analyses were done using SPSS software version 21. P values < 0.05 were considered statistically significant.

### Ethical considerations

This study has been approved by Local ethics review boards at Shahid Beheshti University of Medical Sciences, Tehran, Iran (ir.sums.rec.1395.100).

## Results

About 65% (n = 248) of the participants had one or two copies of the risk allele. General characteristics of the participants with different FTO genotypes were not significantly different (P > 0.05) (Table 1). Regarding to lipid profile, the lower levels of HDL were observed in AT/AA genotypes compared to the TT wild type genotype of FTO rs9939609 polymorphism (P < 0.05) (Table 1).

**Table 1:** General characteristics of the participants based on their FTO genotype

	TT (n = 127)	AT/AA (n = 248)	P
Age (yr)	51.51 (± 9.79)	51.53 (9.72)	0.98
Height (cm)	156.34 (± 5.08)	156.617(± 6.23)	0.66
Weight (kg)	71.27 (± 12.53)	71.08 (± 11.39)	0.89
BMI (kg/m^2)^	29.10 (± 4.630)	28.95(± 4.30)	0.77
Physical activity (min/wk)	161 (± 15.6)	151 (± 24.6)	0.64
TG (mg/dl)	121.97 (± 69.6535)	126.791(± 64.02)	0.56
Chol (mg/dl)	201.02 (± 39.1499)	192.155(± 38.37)	0.06
HDL-C (mg/dl)	57.53 (± 9.5037)	54.79 (± 11.85)	0.03
LDL-C(mg/dl)	1.19 (± 32.78)	1.12 (± 32.89)	0.07
Calorie intake (Kcal)	2600 (± 1.01)	2570 (± 1.10)	0.88
Protein (mg/dl)	8.43 (± 37.10)	8.71 (± 49.08)	0.75
Total fat (kg)	9.64 (± 48.44)	9.24 (± 52.40)	0.70

*BMI* body mass index, *TG* triglyceride, *Cho* cholesterol

Table 2 presented the percentages of the participants with low and high levels of lipid profile based on their FTO genotype. The number of people with higher HDL level was significantly higher in TT FTO genotype group compared to the AT/AA genotypes (79% vs 62%; P = 0.004). Regarding to levels of TG, Chol, and LDL, no significant difference was found between different genotypes of FTO gene (all P > 0.05) (Table 2).

**Table 2:** Lipid profile of the participants based on their FTO genotype

	TT (n = 127)	AT/AA (n = 248)	P
TG			0.52
≤ 150	77 (74%)	143 (74.5%)	
150 <	27 (26%)	49 (25.5%)	
Cholesterol			0.32
≤ 200	56 (53.8%)	116 (60.4%)	
200 <	48 (46.2%)	76 (39.6%)	
LDL			0.16
≤ 100	30 (28.8%)	72 (37.50%)	
100 <	74 (71.2%)	120 (62.5%)	
HDL			0.004
≤ 50	22 (21.2%)	73 (38.0%)	
50 <	82 (78.8%)	119 (62%)	

*TG* triglyceride, *LDL* law density cholesterol, *HDL* high density cholesterol

Logistic regression method identified a negative association between the risk allele of FTO rs9939609 polymorphism and high levels of serum HDL (Table 3, model 1). Adjustment of confounding variables including age and BMI did not change the results (Table 3, model 2). The association remained significant after further adjustments for physical activity (Table 3, model 3). Interestingly, further adjustments for dietary intake including the intake of calorie, carbohydrate, protein, and fat disappeared the significant association between FTO genotype and the HDL level (Table 3, model 4).

**Table 3:** Logistic regression for the association between HDL-C and FTO genotype

	TG		Chol		LDL		HDL	
	OR (CI95%)	P	OR (CI95%)	P	OR (CI95%)	P	OR (CI95%)	P
Model 1	1.023 (0.59–1.76)	0.934	1.308 (0.81–2.12)	0.275	1.480 (0.88–2.48)	0.136	2.28 (1.31–3.97)	0.003
Model 2	0.979 (0.55–1.73)	0.941	1.307 (0.79–2.15)	0.275	1.47 (0.88–2.49)	0.14	2.42 (1.37–4.27)	0.002
Model 3	0.867 (0.48–1.55)	0.633	1.250 (90.75–2.08)	0.391	1.50 (0.87–2.56)	0.13	2.47 (1.39–4.01)	0.002
Model 4	1.549 (0.39–6.08)	0.531	1.991 (0.61–6.47)	0.253	2.65 (0.61–11.47)	0.19	1.65 (0.34–8.11)	0.53

*Model 1* crude, *Model 2* Adjusted for age and BMI, *Model 3* Adjusted for age, BMI, and physical activity, *Model 4* Adjusted for age, BMI, physical activity, and diet

## Discussion

The results of this study indicated that the number of people with TT FTO genotype group had higher HDL levels compared to the AA/AT carriers. No significant differences were found between different genotypes of FTO gene regarding to the levels of TG, Chol, and LDL. Adjusting for potential cofounding variables including age, BMI, and physical activity did not change the results. However, the significant result was disappeared after further adjustment for dietary intake. It indicated that the association between the FTO genotype and serum HDL levels might be affected by dietary intake.

Several studies were carried out to demonstrate the association between FTO genotype and lipid profile. In line with this study, Franczak et al. reported that there is no association between FTO gene polymorphisms and BMI, total cholesterol, LDL cholesterol, and triglyceride. While, the FTO risk alleles were associated with decreased HDL cholesterol concentration. Homozygotes for the rs9939609 risk allele had 1.27-fold lower HDL cholesterol concentration than carriers of the TT genotype [28]. In addition, Khella et al. demonstrated that there was no significant difference in both anthropometric and biochemical measurements of the participants with different FTO rs9939609 genotypes at all genetic models (additive, dominant, and recessive) except for HDL-C levels. The AA genotype carriers had significantly lower levels of HDL-C [29]. Zhang et al. also reported that carriers of the A-allele of rs9939609 polymorphism had lower HDL-c compared with the controls [30]. In contrast with our results, one studies found no significant association between FTO polymorphisms and HDL-c level [31]. However, this study was carried out on children. It is possible that the association between FTO genotype and serum HDL-c level is influenced by ages. Another study in Pakistan also found no association between the lipid profile parameters and rs9939609 polymorphism of FTO gene [32]. Moraes et al. reported FTO gene is associated with LDL but not with HDL cholesterol in obese people that can imply a possible role for obesity in the association between FTO genotype and lipid profile [33]. HDL-c level is known to be associated with metabolic diseases. Based on the findings of this study, carriers of A-allele of FTO rs9939609 polymorphism had lower levels of HDL-c and might be more susceptible to metabolic and cardiovascular diseases.

Moreover, the FTO gene belongs to the superfamily of Fe (II)- and 2-oxoglutarate-dependent dioxygenases which plays a key role in demethylation of nucleic acids and transfected FTO localizes to the nucleus [34, 35]. These biological properties of FTO suggest the possibility that FTO may regulate the expression of other genes through modification of their methylation–demethylation states. It is therefore proposed that FTO plays a role in the regulation of metabolism, possibly by altering the expression level of other genes in metabolically active tissues [36]. The interaction of FTO with calmodulin-dependent protein kinase II (CaMKII) triggers the prolongation of CREB phosphorylation, which ultimately affects the expression levels of Brain-derived neurotrophic factor (BDNF) and NPY1R neuropeptide Y receptor Y1 (NPY1R) that regulate energy balance and they are reported to be related with lipid metabolic process [22].

One of the strengths of this study was considering various confounders in statistical analysis to obtain more accurate results. The present study reported significant association between risk allele of FTO rs9939609 polymorphism and HDL-c after adjustment for age, weight, height, physical activity and BMI. However, dietary intake changed the association between HDL and FTO gene. Since FTO gene is widely expressed in several tissues such as brain, visceral fat, liver, and hypothalamus, FTO gene variants can play important roles in appetite regulation, food intake and tendency to choose high fat and high carbohydrate diet [37, 38].

However, this study had some limitations. The association of different genotypes of FTO rs9939609 polymorphism with lipid profile was investigated only in women. The associations of this polymorphism with lipid profile can be different across various ethnicities and age groups. Therefore, it is strongly recommended to carry out future studies across different populations in different ages and genders with a wide spectrum of BMI to better understand the associations and effect sizes.

## Conclusion

These results strongly indicated an association between HDL-c and FTO genotype. Adjusting for potential cofounding variables including age, BMI, and physical activity did not change the results. However, the association between the FTO genotype and serum HDL levels was affected by dietary intake. Further longitudinal studies are needed to determine the association between FTO genotype and lipid profile to identify the underlying mechanisms.

## Data Availability

Not applicable.

## Abbreviations

TG: Triglycerides
TC: Total cholesterol
HDL-C: High-density lipoprotein cholesterol
LDL-C: Low-density lipoprotein cholesterol
